# Balo Concentric Sclerosis: A Rare Variant of Multiple Sclerosis With Excellent Response to Early Steroid Treatment

**DOI:** 10.7759/cureus.86059

**Published:** 2025-06-15

**Authors:** Zhuo Luan, Khan Z Habiba, Amy H Sim, Isabel V Narvaez-Correa

**Affiliations:** 1 Neurology, Texas Tech University Health Sciences Center El Paso, EL Paso, USA; 2 Radiology, Texas Tech University Health Sciences Center El Paso, El Paso, USA; 3 Neurology, Texas Tech University Health Sciences Center El Paso, El Paso, USA

**Keywords:** balo concentric sclerosis (bcs), corticosteroid treatment, early diagnosis and intervention, ms (multiple sclerosis), neuroimaging studies

## Abstract

Balo concentric sclerosis (BCS) is a rare and aggressive variant of multiple sclerosis (MS), characterized by unique alternating concentric layers of demyelinated and preserved myelin on neuroimaging. We present a case of a 34-year-old male patient with progressive right-sided weakness, whose MRI brain findings confirmed BCS. The patient demonstrated rapid clinical improvement with early initiation of high-dose corticosteroids. The Expanded Disability Status Scale (EDSS) score improved from 6 to 1 within one month, underscoring the importance of timely intervention. Early recognition and prompt treatment are critical to improving outcomes in BCS, as delays can lead to irreversible neurological damage. This case highlights the significant role of early high-dose corticosteroid therapy in achieving clinical improvement and preventing long-term disability.

## Introduction

Multiple sclerosis (MS) is a long-standing, immune-driven demyelinating disorder of the central nervous system, impacting an estimated 2.8 million individuals worldwide, with a global prevalence of approximately 35.9 cases per 100,000 population [1]. Balo concentric sclerosis (BCS) is a rare demyelinating disease that falls under the spectrum of MS but is distinguished by its unique radiological and pathological features. It is primarily diagnosed based on its characteristic "onion bulb" or "concentric ring" appearance on MRI, which represents alternating layers of demyelination and preserved myelin. First described by József Balo in 1928, BCS remains an infrequent but critical differential diagnosis for atypical MS presentations [2,3]. Owing to its exceptional rarity, BCS has no well-defined incidence or prevalence figures at the population level.

BCS is often misdiagnosed due to its rarity and overlapping features with other demyelinating diseases, such as tumefactive MS, Marburg variant MS, and even malignancies. The hallmark MRI finding of concentric rings of demyelination is essential for accurate diagnosis [4]. Early identification and treatment are crucial, as delays can lead to irreversible neurological damage and long-term disability [5,6]. This case highlights early high-dose corticosteroid therapy in achieving clinical improvement and preventing long-term disability.

## Case presentation

A 34-year-old Hispanic male patient with no significant past medical history presented with progressive right-sided weakness that began eight days prior to admission. The patient first noticed right lower extremity numbness and mild weakness without any difficulty ambulating. Four days later, he developed right upper extremity numbness, prompting him to seek medical attention. He was admitted to a local hospital from day 5 to day 7, where initial imaging suggested demyelination or tumor. The clinical team debated whether the patient’s symptoms were due to a tumor or a demyelinating disorder. A lumbar puncture (LP) was planned to further investigate the etiology, but the patient decided to seek a second opinion and was discharged without undergoing the procedure. Due to persistent symptoms, he presented to our facility for further evaluation.

**Figure 1:**
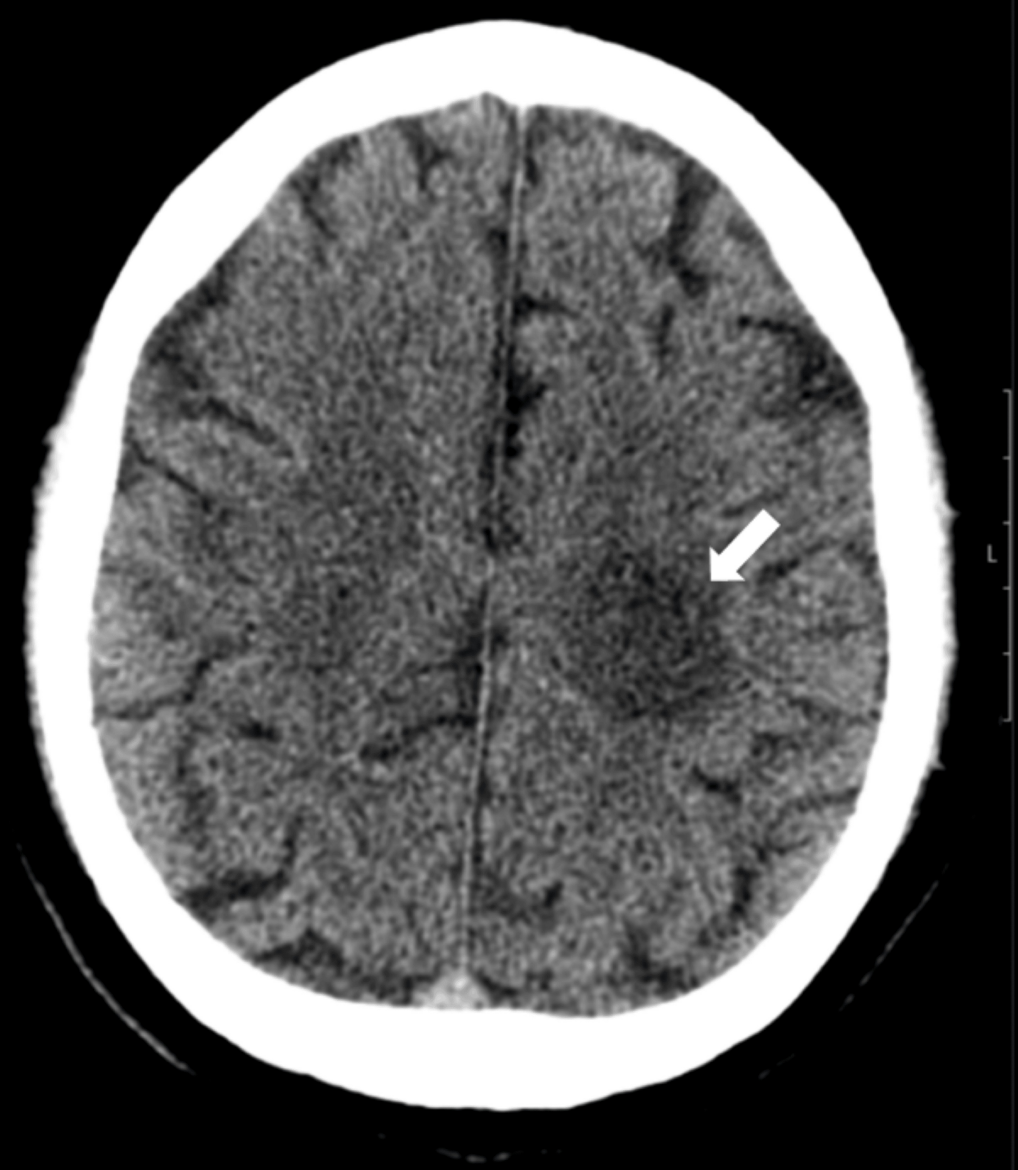
Non-contrast CT of the head. Axial slice shows a rounded hypodensity in the left subcortical superior parietal lobule (arrow), consistent with a focal lesion.

On physical examination, the patient appeared well and was in no acute distress. Neurological examination revealed right proximal upper extremity strength of 3/5 with distal wrist drop (2/5) and right lower extremity strength of 4/5. He had difficulty walking and required assistance. Strength in the left upper and lower extremities was normal. Sensation to light touch, pain, temperature, and proprioception was intact, with normal reflexes throughout. The Expanded Disability Status Scale (EDSS) score was 6. Imaging studies included head CT (Figure 1), which revealed a hypodensity in the left centrum semiovale sparing the cortex.

**Figure 2:**
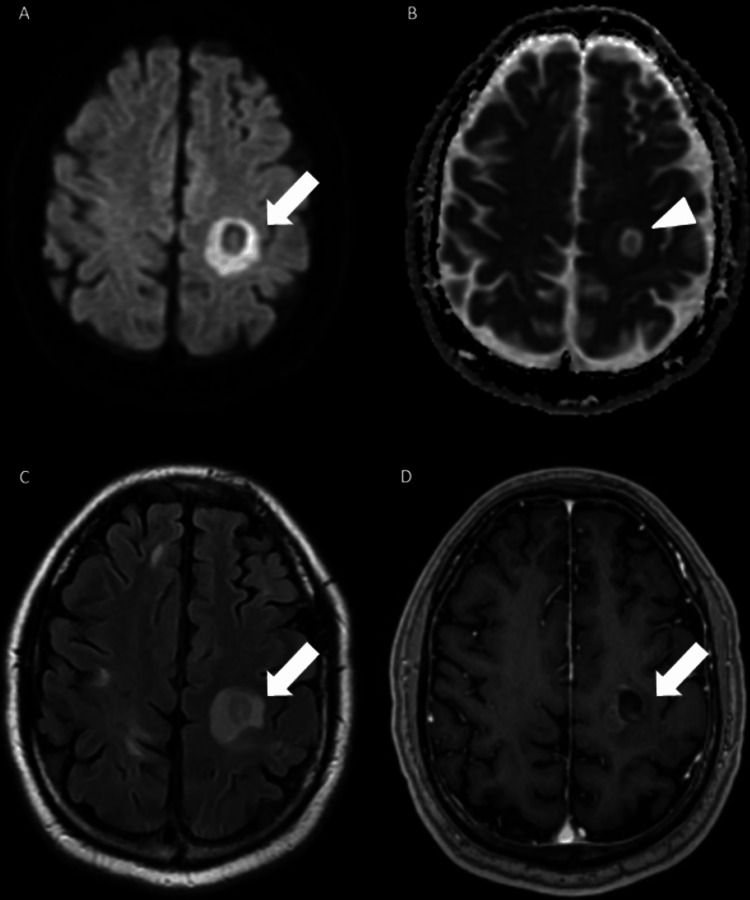
Axial MRI brain sequences. The lesion corresponding to the CT hypodensity demonstrates diffusion restriction on DWI (A), T2 hyperintensity (C), and heterogeneous open-ring enhancement on post-contrast T1-weighted imaging (D), all marked by arrows. On the ADC map (B), a layered “onion peel” appearance is observed (arrowhead), which is faintly discernible on other sequences. No significant mass effect is present despite the lesion’s size. DWI: diffusion-weighted imaging; ADC: apparent diffusion coefficient.

**Figure 3:**
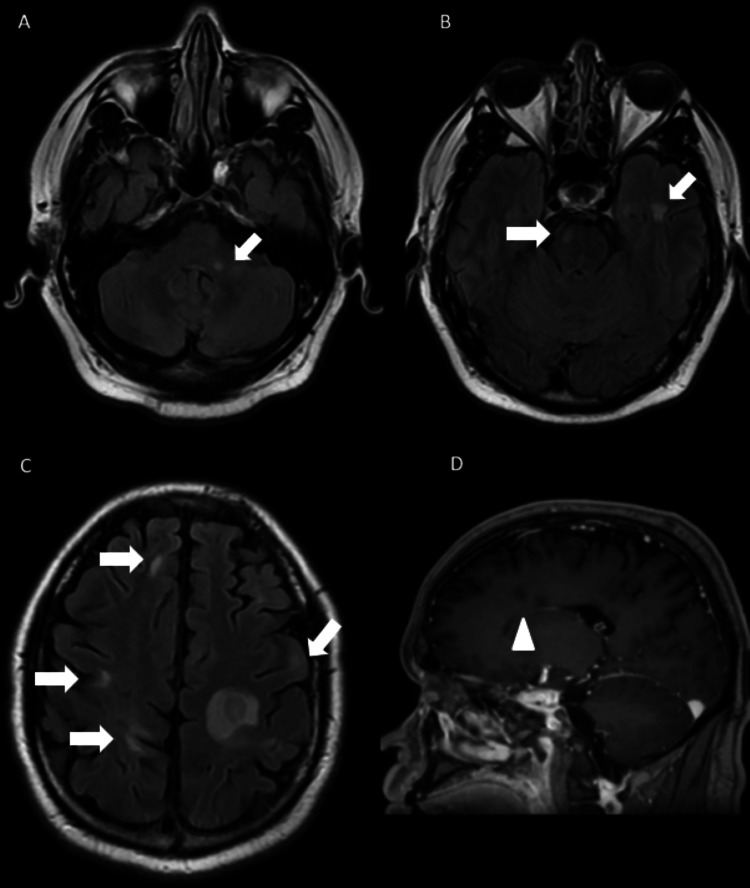
Additional MRI brain images. Axial T2 FLAIR images through the pons (A), midbrain (B), and cerebrum (C) demonstrate scattered supra- and infratentorial subcortical and deep white matter hyperintensities involving the left middle cerebellar peduncle, right ventral pons, left anterior temporal lobe, and right frontal and parietal lobes (arrows). A sagittal post-contrast T1-weighted image (D) shows periventricular hypointense lesions consistent with classic "Dawson’s fingers" (arrowhead). FLAIR: fluid-attenuated inversion recovery.

MRI of the brain (Figures 2, 3) demonstrated a lesion with diffusion restriction, T2 hyperintensity, and heterogeneous open ring enhancement on post-contrast T1 imaging. The apparent diffusion coefficient (ADC) map revealed a distinctive layered ‘onion peel’ pattern, which is characteristic of BCS.

**Figure 4:**
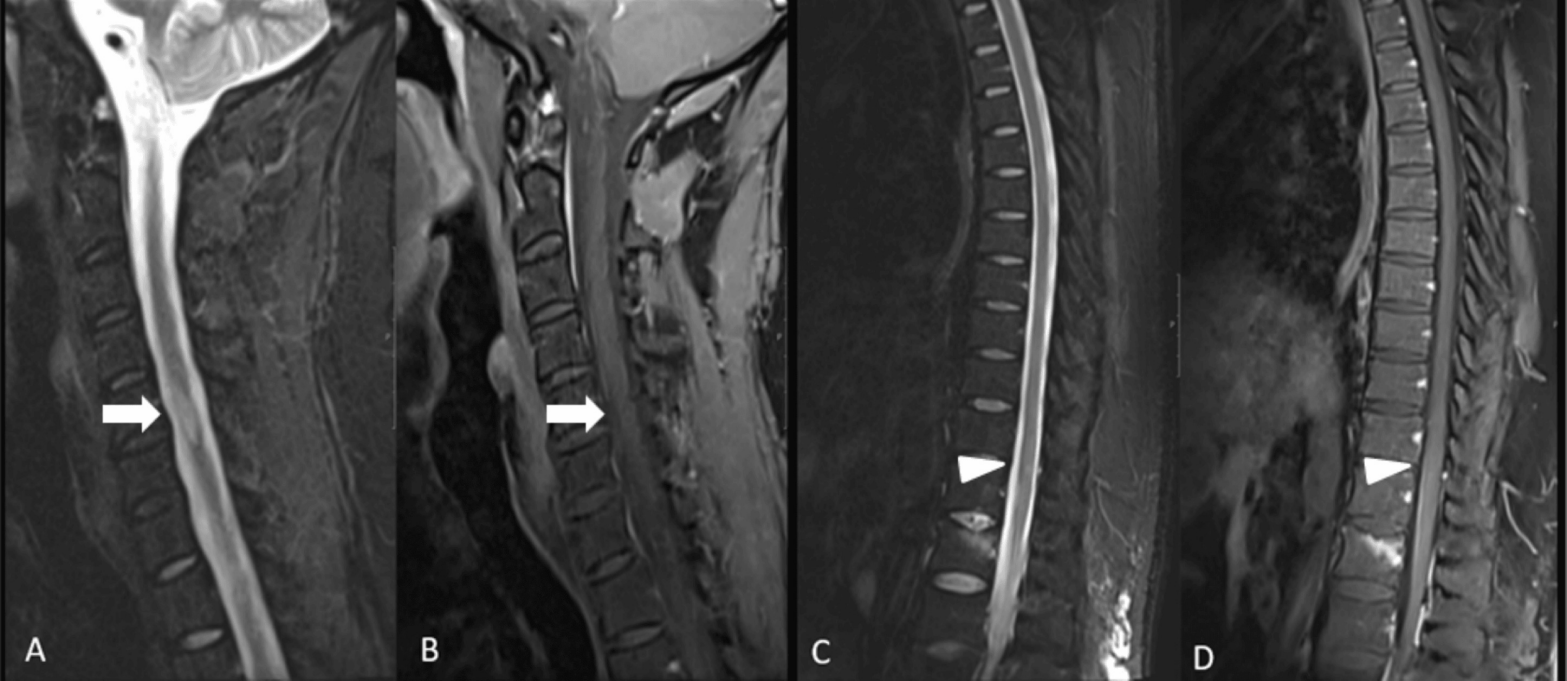
MRI of the cervical and thoracic spine. STIR images (A, C) and post-contrast T1-weighted images (B, D) reveal a non-enhancing demyelinating lesion in the peripheral spinal cord at the C5–C6 level (arrow in A; A-B). Additionally, subtle patchy lesions are observed throughout the thoracic spinal cord (arrowhead in C; C-D), without contrast enhancement or cord expansion. STIR: short tau inversion recovery.

MRI of the spine (Figure 4) showed multiple patchy T2 STIR (Short Tau Inversion Recovery) hyperintensities in the thoracic cord, notably at T4, T6, T8, and T12, as well as at the conus, with no enhancing lesions identified.

The patient was promptly started on high-dose IV methylprednisolone 1 g/day for 3 days. By day 3, the patient showed significant improvement with corticosteroid therapy. He was able to walk without difficulty and had right proximal upper extremity strength of 4/5, and right lower extremity strength of 5/5. The EDSS score was 2, and he was discharged with a one-week methylprednisolone taper. He was scheduled for a follow-up in the neurology clinic.

At the four-week follow-up, the patient showed significant improvement. On follow-up, he reported mild residual clumsiness in the right hand but denied any new weakness or vision changes. Physical examination revealed intact cranial nerves, normal sensation, and mild right upper extremity weakness (4+/5 strength in arm abduction, 5/5 in other muscle groups), EDSS 1. The patient was educated about MS pathophysiology and treatment options, agreed to start Ocrevus (ocrelizumab), and was scheduled for follow-up in three months. Overall, the patient demonstrated significant clinical improvement with residual mild right arm weakness, and long-term disease-modifying therapy was initiated to prevent further relapses.

## Discussion

BCS is a rare and aggressive demyelinating disorder characterized by its distinctive MRI findings of concentric rings. The patient’s rapid clinical improvement following early high-dose corticosteroid therapy underscores the critical role of timely intervention in BCS. Early treatment not only halts disease progression but also significantly reduces the risk of long-term disability [7], as demonstrated in this case. For steroid-refractory cases, plasma exchange (PLEX) may be considered as a second-line treatment. Long-term disease-modifying therapies, such as ocrelizumab, rituximab, and natalizumab, are often used to prevent relapses and disease progression [8,9].

Our review of published case reports and series on BCS indicates that patients most often present between 18 and 41 years of age with sudden neurological symptoms such as unilateral weakness, language impairment, seizures, or visual changes. The disease course varies, ranging from a single episode to relapsing or steadily worsening forms. While the majority of patients show significant improvement with high-dose corticosteroid therapy, a subset requires additional immunomodulatory or disease-modifying interventions. Generally, the outlook is favorable, with many individuals experiencing full or partial recovery, although isolated cases of fatal outcomes have been documented [7,10,11].

The differential diagnosis for BCS includes tumefactive MS, Marburg variant MS, and malignancies [12,13]. Additionally, BCS can be associated with other conditions that affect the central nervous system, including ADEM (Acute Disseminated Encephalomyelitis), neuromyelitis optica spectrum disorders, PML (Progressive Multifocal Leukoencephalopathy), and CADASIL (Cerebral Autosomal Dominant Arteriopathy with Subcortical Infarcts and Leukoencephalopathy) [14,15]. These associations highlight the importance of considering a broad differential diagnosis, as distinguishing BCS from these entities is essential for guiding appropriate treatment and prognosis. The distinctive “onion bulb” or “bullseye” pattern on MRI plays a key role in differentiating BCS from other similar conditions [16]. Misdiagnosis can lead to unnecessary invasive procedures and delays in appropriate treatment, further emphasizing the importance of awareness of BCS imaging features.

The pathogenesis of BCS, though not fully understood. Studies suggest that alternating concentric rings of demyelination and preserved myelin may arise due to inflammatory mediators, hypoxia, or metabolic stress [17]. Specifically, oligodendrocyte injury caused by an excessive immune response may trigger alternating zones of demyelination and partial remyelination, resulting in the classic "onion bulb" appearance on MRI [18]. Emerging therapies, such as Bruton's tyrosine kinase (BTK) inhibitors and remyelination agents, are currently under investigation and may offer new treatment options for patients with BCS in the future [19,20].

## Conclusions

This case underscores the importance of considering Balo concentric sclerosis in atypical demyelinating presentations. Given its potential for rapid progression, early recognition and aggressive treatment are paramount. The patient’s excellent response to early high-dose corticosteroid therapy highlights the critical role of timely intervention in improving outcomes. Further research is needed to refine management strategies and long-term prognosis.

## References

[1] Walton C, King R, Rechtman L (2020). Rising prevalence of multiple sclerosis worldwide: insights from the Atlas of MS, third edition. Mult Scler.

[2] Hardy TA, Miller DH (2014). Balo's concentric sclerosis. Lancet Neurol.

[3] Balo J (1928). Encephalitis periaxialis concentrica. Arch NeurPsych.

[4] Xie JS, Jeeva-Patel T, Margolin E (2021). Baló's concentric sclerosis - a rare entity within the spectrum of demyelinating diseases. J Neurol Sci.

[5] Ontaneda D, Chitnis T, Rammohan K, Obeidat AZ (2024). Identification and management of subclinical disease activity in early multiple sclerosis: a review. J Neurol.

[6] Jolliffe EA, Guo Y, Hardy TA, Morris PP, Flanagan EP, Lucchinetti CF, Tobin WO (2021). Clinical and radiologic features, pathology, and treatment of baló concentric sclerosis. Neurology.

[7] Martinez HR, Rodriguez-Gonzalez IC, Escamilla-Garza JM, Figueroa-Sanchez JA, Garcia-Aleman AC, Hinojosa-Gonzalez DE (2021). Balo's Concentric Sclerosis with monophasic course: a report of 2 cases. Ann Med Surg (Lond).

[8] Keegan M, Pineda AA, McClelland RL, Darby CH, Rodriguez M, Weinshenker BG (2002). Plasma exchange for severe attacks of CNS demyelination: predictors of response. Neurology.

[9] Gibiansky E, Petry C, Mercier F (2021). Ocrelizumab in relapsing and primary progressive multiple sclerosis: pharmacokinetic and pharmacodynamic analyses of OPERA I, OPERA II and ORATORIO. Br J Clin Pharmacol.

[10] Tzanetakos D, Vakrakou AG, Tzartos JS (2020). Heterogeneity of Baló's concentric sclerosis: a study of eight cases with different therapeutic concepts. BMC Neurol.

[11] Etemadifar M, Aghili A, Shojaei S, Alaei SA, Salari M, Norouzi M (2025). Balo concentric sclerosis, an emerging variant of multiple sclerosis: A case-series and literature review. J Neuroimmunol.

[12] Capello E, Mancardi GL (2004). Marburg type and Balò's concentric sclerosis: rare and acute variants of multiple sclerosis. Neurol Sci.

[13] Amini Harandi A, Esfandani A, Pakdaman H, Abbasi M, Sahraian MA (2018). Balo's concentric sclerosis: an update and comprehensive literature review. Rev Neurosci.

[14] Chitnis T, Hollmann TJ (2012). CADASIL mutation and Balo concentric sclerosis: a link between demyelination and ischemia?. Neurology.

[15] Jarius S, Würthwein C, Behrens JR, Wanner J, Haas J, Paul F, Wildemann B (2018). Baló's concentric sclerosis is immunologically distinct from multiple sclerosis: results from retrospective analysis of almost 150 lumbar punctures. J Neuroinflammation.

[16] Ng SH, Ko SF, Cheung YC, Wong HF, Wan YL (1999). MRI features of Balo's concentric sclerosis. Br J Radiol.

[17] Stadelmann C, Ludwin S, Tabira T (2005). Tissue preconditioning may explain concentric lesions in Baló's type of multiple sclerosis. Brain.

[18] Nakayama M, Naganawa S, Ouyang M, Jones KA, Kim J, Capizzano AA, Moritani T (2021). A review of clinical and imaging findings in tumefactive demyelination. AJR Am J Roentgenol.

[19] Greenberg BM (2024). Bruton's tyrosine kinase inhibitors for multiple sclerosis treatment: a new frontier. Neurol Clin.

[20] Bourdette D, Wooliscroft L (2024). Developing drugs that promote remyelination: Is our in vitro screening approach too simplistic?. Neurotherapeutics.

